# Novel needle system for transmural access in therapeutic endoscopic ultrasound

**DOI:** 10.1055/a-2760-9144

**Published:** 2026-01-22

**Authors:** Stefano Mazza, Davide Scalvini, Aurelio Mauro, Marco Bardone, Daniele Alfieri, Francesca Torello Viera, Andrea Anderloni

**Affiliations:** 1 18631 Gastroenterology and Digestive Endoscopy Unit, Fondazione IRCCS Policlinico San Matteo, Pavia, Italy; 2 19001 Department of Internal Medicine and Medical Therapeutics, University of Pavia, Pavia, Italy

Endoscopic ultrasound (EUS)-guided transmural access has expanded dramatically over the last decade and now plays a pivotal role in biliary drainage as an alternative to endoscopic retrograde cholangiopancreatography (ERCP 1 2 ). Electrocautery-enhanced lumen apposing metal stents with the free-hand technique, which allow for a single-step, single-device procedure, have strongly contributed to such an expansion, although many procedures still require a Seldinger approach involving access with needle puncture and guidewire positioning in the biliary system 2 3 4 . However, these are demanding techniques and dedicated devices that facilitate the technical execution of the procedure are lacking.

Three patients aged between 68 and 84 years presented with malignant biliary obstruction, two located distally and one perihilar, treated with EUS-guided biliary drainage after ERCP failure. All procedures required the “classic” approach, for which a new EUS-guided access needle device specifically designed for transmural access was used (SonoTip AccessPro, Medi-Globe, Rohrdorf, Germany). The device is shown in detail in Video 1 .

The SonoTip AccessPro needle for EUS-guided transmural access is introduced, and its use during an EUS-guided hepaticogastrostomy is shown. EUS, endoscopic ultrasound.Video 1

The perihilar obstruction was due to a large metastatic lymphadenopathy, with ERCP not feasible because of duodenal infiltration at the duodenal neck. In this case, endoscopic ultrasound-guided hepaticogastrostomy was performed. After accessing the B2 segment intrahepatic bile ducts from the stomach, the guidewire was advanced beyond the obstruction into the common bile duct. A dedicated partially covered metal stent was placed ( Video 1 ).

In the two cases of distal obstruction, papillary cannulation was not possible, and EUS-guided biliary drainage was not feasible because of insufficient bile duct dilation. Thus, EUS-guided rendezvous ERCP was performed, in one case with access from the common bile duct and side-by-side papillary cannulation ( Fig. 1 ) and in the other case with access from the left intrahepatic bile ducts and over-the-wire cannulation ( Fig. 2 ). A transpapillary fully covered metal stent was placed in both cases.

**Fig. 1:**
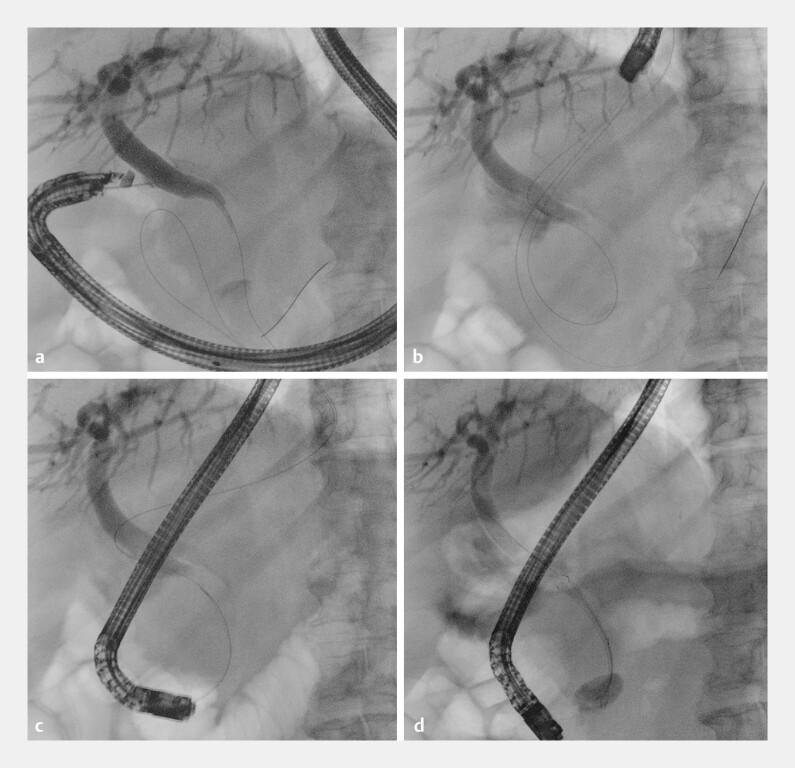
**a** EUS-guided access to the common bile duct and guidewire advancement through the papilla into the duodenal lumen. **b** Endoscopic retrieval of the guidewire end exiting from the papilla. **c** Over-the-guidewire cannulation. **d** Transpapillary biliary stent deployment. EUS, endoscopic ultrasound.

**Fig. 2:**
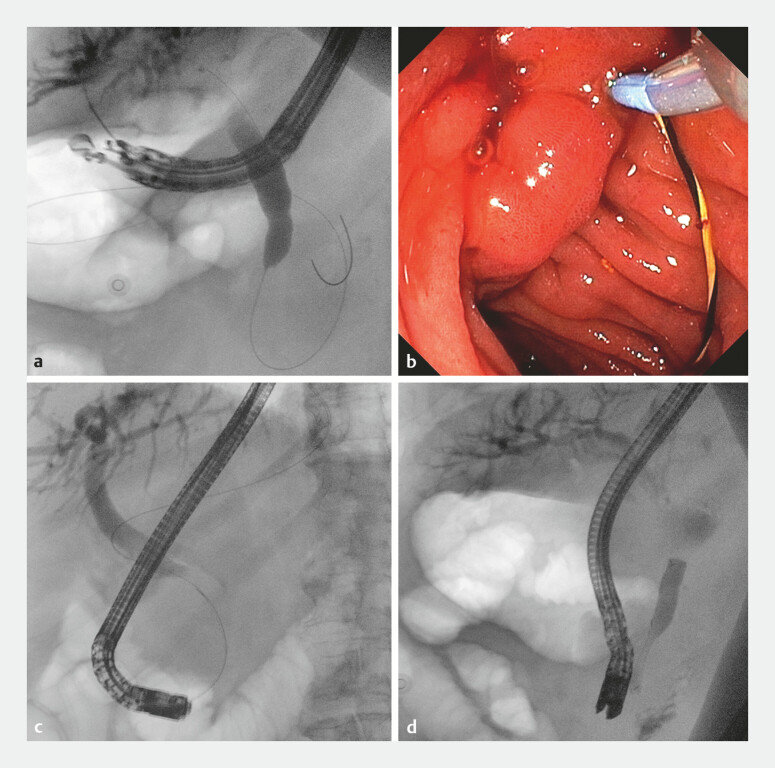
**a** EUS-guided access to the left intrahepatic bile duct and guidewire advancement through the papilla into the duodenal lumen. **b** An endoscopic view of side-by-side cannulation. **c** A Fluoroscopic view of side-by-side cannulation and retrograde guidewire positioning. **d** Transpapillary biliary stent deployment. EUS, endoscopic ultrasound.

Endoscopy_UCTN_Code_TTT_1AS_2AD

## Notes

Endoscopy E-Videos https://eref.thieme.de/e-videos
*E-Videos* is an open access online section of the journal *Endoscopy* , reporting on interesting cases and new techniques in gastroenterological endoscopy. All papers include a high-quality video and are published with a Creative Commons CC-BY license. Endoscopy E-Videos qualify for HINARI discounts and waivers and eligibility is automatically checked during the submission process. We grant 100% waivers to articles whose corresponding authors are based in Group A countries and 50% waivers to those who are based in Group B countries as classified by Research4Life (see: https://www.research4life.org/access/eligibility/ ). This section has its own submission website at https://mc.manuscriptcentral.com/e-videos .
